# Modelling immune responses in sepsis

**DOI:** 10.1186/cc10611

**Published:** 2012-03-20

**Authors:** R Grealy, M White, M O'Dwyer, P Stordeur, DG Doherty, R McManus, T Ryan

**Affiliations:** 1 Trinity College Dublin, Ireland; 2 St James's Hospital, Dublin, Ireland; 3 Hopital d'Erasme, Bruxelles, Belgium

## Introduction

The onset and evolution of the sepsis syndrome in humans is modulated by an underlying immune suppressive state [1,2]. Signalling between immune effector cells plays an important part in this response. The objective of this study was to investigate peripheral blood cytokine gene expression patterns and serum protein analysis in an attempt to model immune responses in patients with sepsis of varying severity. We hypothesised that such immunologic profiling could be of use in modelling and prediction of outcomes in sepsis in addition to the evaluation of future novel sepsis therapies.

## Methods

A prospective observational study in a mixed medical/surgical ICU and general wards of a large academic teaching hospital was undertaken. Eighty ICU patients with a diagnosis of severe sepsis, 50 patients with mild sepsis (bacteraemia not requiring ICU admission) and 20 healthy controls were recruited. Gene expression analysis by qPCR for INFγ, TNFα, IL-2, IL-7, IL-10, IL-23, IL-27 on peripheral blood mononuclear cells (PBMCs) and serum protein analysis for IL-6 was performed. Multivariate analysis was used to construct a model of gene expression based on cytokine copy numbers alone and in combination with serum IL-6 levels.

## Results

Sepsis was characterised by decreased IL-2, IL-7, IL-23, INFγ and greater TNFα, IL-10 and IL-27 gene expression levels compared to controls. Severe sepsis differed from mild sepsis by a decreased INFγ and increased IL-10 gene expression (*P *< 0.0001). A composite cytokine gene expression score differentiated controls from mild sepsis and mild sepsis from severe sepsis (*P *< 0.0001). A model combining these cytokine gene expression levels and serum IL-6 protein levels distinguished sepsis from severe sepsis with an ROC value of 0.89.

## Conclusion

Accurate modelling of patient response to infection is possible using peripheral blood mononuclear cell gene expression and serum protein analysis. Molecular biological techniques provide a robust method of such profiling. This approach may be used to evaluate novel sepsis therapies.
